# Short-Term (4 Day) Effects of Oral Rinsing with Miswak and Green Tea on Gingival Crevicular Fluid Flow and IL-1β Levels: A Pilot Study

**DOI:** 10.3390/healthcare11020226

**Published:** 2023-01-11

**Authors:** Rasha Salah, Hayder Raad Abdulbaqi

**Affiliations:** Department of Periodontics, College of Dentistry, University of Baghdad, Baghdad 10071, Iraq

**Keywords:** Salvadoraceae, tea, gingival crevicular fluid, interleukin-1β, mouthwashes

## Abstract

Despite the antiplaque effect of mouth-rinsing with a combination composed of miswak (*Salvadora persica* L.) and green tea (*Camellia sinensis* var. *assamica*) extracts, no data are available regarding its effect on gingival tissue at the molecular level. This pilot study aimed to assess the effect of oral rinsing with this combination on gingival crevicular fluid (GCF) flow and IL-1β levels. Ten subjects rinsed with either the combination, 0.12% chlorhexidine gluconate (CHX) or distilled water without toothbrushing for 4 days after receiving baseline polishing. GCF IL-1β concentration, influx, resting volume and plaque quantity were measured at baseline and after 4 days for each intervention. No significant differences in GCF flow or resting volume were detected after rinsing with the different mouthwashes. A significant increase in GCF IL-1β concentration was evident only after rinsing with distilled water. Rinsing with combination induced a significant reduction in GCF influx (−0.086 ± 0.222) compared to CHX (0.088 ± 0.247) and distilled water (0.075 ± 0.201). Less plaque was detected after rinsing with combination and CHX. Short-term oral rinsing with this combination could potentially induce no significant changes in GCF flow and IL-1β concentration, and might retard inflammation. Thus, it might be considered in the production of natural oral healthcare products.

## 1. Introduction

Gingival crevicular fluid (GCF) is a clear watery liquid that leaks into healthy gingival sulcus and periodontal pockets from subepithelial gingival tissue through junctional epithelium and flows into the oral cavity [1]. GCF contains a mixture of substances derived from oozed serum, inflammatory cells and mediators, and host tissue elements, in addition to bacteria and its components such as endotoxins. The flow rate and volume of GCF are clinical parameters that can be noninvasively determined. Their magnitudes are directly proportional to gingival tissue inflammation and have been suggested to be indicators of gingival inflammation. Moreover, the levels of many proinflammatory mediators in GCF have been proposed to be sensitive diagnostic tools that can help to precisely indicate the severity of periodontal diseases [2,3]. One of these mediators is interleukin-1β (IL-1β), whose level in GCF was reported to increase upon exaggeration of periodontal inflammation and to decline in response to treatment [4,5]. IL-1β is a proinflammatory cytokine participating in the innate immune response and is mainly secreted from macrophages within gingival tissue in response to bacterial insults [6]. Along with other proinflammatory cytokines, it is essentially involved in the initiation of inflammatory immune reactions and is continually secreted during the inflammatory process [7,8]. After a short period of three days of non-brushing, IL-1β has been found to increase to a significant level in GCF upon the accumulation of dental biofilms on tooth surfaces, even before the emergence of any clinical signs of inflammation in the surrounding gingiva [9]. Dental plaque (or dental biofilm) accumulated on tooth surfaces is the principal factor in the initiation and progression of gingival inflammation [10]. As it develops on tooth surfaces, its microbial components elicit defense immune responses in the gingival tissue by stimulating immune cells to produce cytokines; IL-1β is one of the earliest cytokines produced. This gingival inflammation is chronically sustained as dental biofilm is left undisturbed [4,5]. Thus, controlling dental biofilms is crucial in the management of gingival inflammation and could be efficiently achieved by tooth brushing. Unfortunately, some proportions of the population lack the dexterity to perform proper tooth brushing [11]. Hence, the use of chemicals to assist in controlling dental biofilms, such as mouthwashes, is fully considered.

Much research in recent years has focused on the use of plant extracts due to their bioactivity and safety. An example is a mouthwash composed of a combination of miswak (*Salvadora persica* L.) and green tea (*Camellia sinensis* var. *assamica*), which was reported to have synergistic antibacterial and anti-adherence actions against primary plaque colonizers in vitro [12] and to retard dental biofilm development in vivo after short-term oral rinsing [13,14]. Constituents of this combination have been reported to exhibit anti-inflammatory properties [15,16]. Thus, the aim of this pilot study was to assess GCF flow and IL-1β levels after oral rinsing with the combination. It was hypothesized that short term oral rinsing with this combination would have no effect on the GCF flow and IL-1β concentration.

## 2. Materials and Methods

A randomized, double blinded crossover study was conducted among 10 male dental students (19–23 years) in the clinics of College of Dentistry, University of Baghdad from January 2019 to March 2019. The subjects were systemically healthy, had no periodontal diseases or active caries, did not wear orthodontic appliances and had not taken any non-steroidal anti-inflammatory drugs or antibiotics for the previous 4 months. This study was registered in ClinicalTrials.gov (NCT03790904).

Due to the novelty of the combination, the sample size was calculated using data of GCF flow of 0.12% chlorhexidine gluconate (CHX) (mean difference = 0.06, SD = 0.05) from the most relevant study [17] using G*Power software (version 3.1.9.2). Seven subjects were found to be sufficient to detect significant differences at a power of 0.8 and a probability of 0.05. To compensate for any drop out, ten subjects was considered the needed sample size.

CHX mouthwash (KIN ™ Gingival, Spain) and distilled water (control; Z S C de-ionized water, Iraq) were used as positive and negative controls, respectively. The combination mouthwash was composed of aqueous extracts of *Camellia sinensis* var. *assamica* (0.25 mg) and *Salvadora persica* L. (7.82 mg) extracts/1 mL distilled water. Roots of *Salvadora persica* L. (Salvadoraceae; the Peelu tree, growing in Pakistan; NUR-AL-KHAIR (ISO 9001:2015, Pakistan)) and leaves of *Camellia sinensis* var. *assamica* (Theaceae; the tea tree, growing in Sri Lanka; BOH green tea (MSI 500:2009, Malaysia)) purchased from markets were used for preparing the aqueous extracts by maceration as described previously [12] in the chemical engineering Research Laboratory, University of Technology, Baghdad, Iraq. The subjects were asked to rinse with 15 mL of allocated intervention for one min twice daily and to refrain from eating and drinking for 30 min. Blinding of the study was ensured by using identical opaque bottles randomly labeled with number codes (1, 2 and 3). A third party not involved in the study randomly allocated the coded bottles to the interventions and generated a random sequential delivery list using a random number generator (Microsoft Excel 2019). Decoding was allowed at the end of the study.

Before starting the trial, the examiner (R.S.) carried out a training session for the measurements of GCF and plaque index (PI) involving 4 volunteers as described [18]; the results were not considered in the analysis. Then, on another day, the examiner (R.S.) recorded PI measurements for another 4 volunteers two times intermitted by a one-hour time interval for the purpose of calibration. A satisfactory intraexaminer agreement of 0.82 kappa value was obtained.

**Figure 1:**
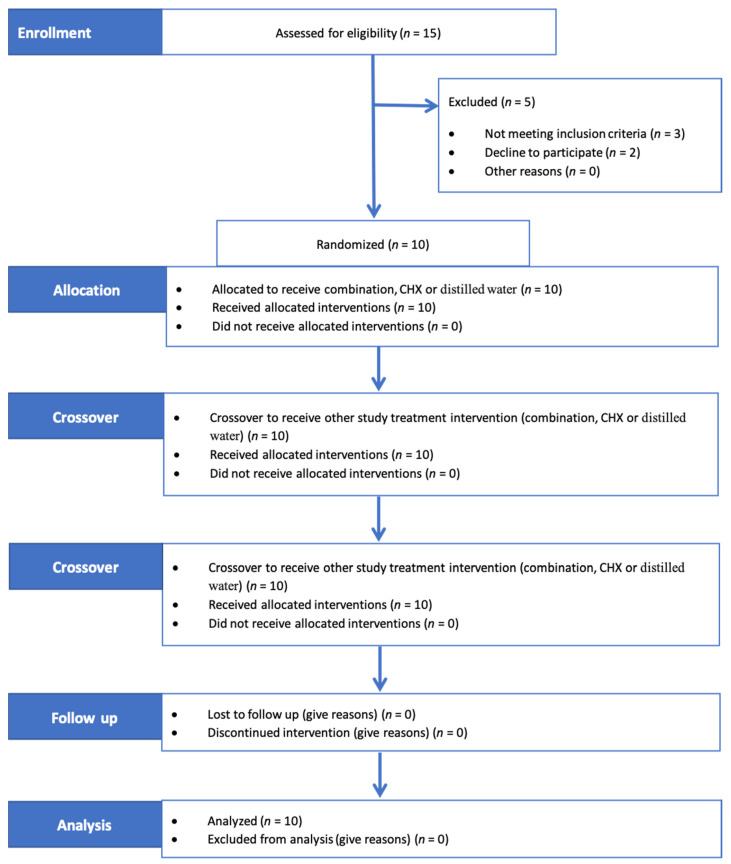
Consort 2010 flow of the study. Subjects rinsed with three mouthwashes through three clinical trial periods intermitted by two washout periods. Combination composed of 7.82 mg/mL miswak (*Salvadora persica* L.) root-stick and 0.25 mg/mL green tea; CHX = 0.12% chlorhexidine.

Following the selection of subjects, all subjects received professional scaling and polishing. After 7 days, a single examiner (R.S.) collected GCF samples before rinsing (T0) from four sites in each subject: the mesiobuccal crevicular region of the cuspid, 1st and 2nd bicuspids and 1st molar of the right upper quadrant. A periopaper strip (ORAFLOW, Smithtown, NY11787) was placed 1–2 mm into the sulcus (after careful drying and isolation), held in place for 30 s and then removed for volume determination (V1). After 30 s, a second periopaper strip was again placed into the site for 3 s (V2). The weight of both strips (V1 and V2) was determined using a sensitive balance (Mettler Toledo ML104, Switzerland) with a precision of 0.0001 g. The flow rate (fi) and resting volume (Vr) of the GCF were measured using the following formulas [19]: (fi = V2/33 s) and (Vr = V1 * 30/fi), respectively. At the mesiobuccal crevicular region of the right upper 1st molar, a periopaper strip was introduced 1–2 mm into the sulcus (after careful drying and isolation) and held in place for 30 s. To avoid blood contamination, the paper strips were only inserted into the sulcus after no clearly visible signs of gingival bleeding were observed. Any strip visibly contaminated with blood was discarded. GCF was eluted by placing the collected strip into Eppendorf tubes containing 200 µL of phosphate-buffered saline (PBS). Then, the tubes were weighed using a sensitive balance and stored at −80 °C until IL-1β measurement [20]. After completion of GCF measurements, each subject was asked to chew an erythrosine tablet for 1 min to disclose the plaque on his teeth surfaces and rinse his mouth with water 3 times. Then, subjects received baseline polishing, and plaque scores were brought to zero. The first mouthwash was given to the subjects, and they were instructed to rinse with 15 mL of the mouthwash for 1 min twice daily for four days and to refrain from all oral hygiene measures during this period. They were also instructed to refrain from eating and drinking for 30 min following each rinsing. At day 4, the plaque index of the designated teeth was recorded using the modified Quigley Hein PI after disclosing the plaque on teeth [21]. GCF samples (T1) and IL1β were again measured. The oral cavity was examined for any possible mouthwash-related adverse effects, such as ulceration, swelling or redness. After that, the subjects entered a 6-day wash-out period. Then, the same protocol was repeated for the next two mouthwashes (see the study flow in Figure 1).

The IL-1β concentration was measured using a commercially available ELISA assay (MYBioSource, Southern California, San Diego, CA, USA), which is based on an antibody sandwich technique. After the designated samples were defrosted at room temperature, wells precoated with a monoclonal antibody specific for human IL-1β were incubated with 100 µL of standard, blank and samples for 1 h at 37 °C. After removing the liquid from each well, 100 µL of detection reagent A working solution was added to each well and incubated for another 1 h. Then, after washing, 100 µL of detection reagent B working solution was added to each well and incubated for 30 min. After additional washing, 90 µL of substrate solution was added to each well for 10 min and incubated at 37 °C. Finally, 50 µL of stop solution was added to each well and the optical density (OD) was read at 450 nm. The assay has a detection sensitivity of less than 5.8 pg/mL.

The data were analyzed using Statistical Package of Social Science (SPSS) version 22.0 for Windows. The Shapiro–Wilk test was used to check the normality of the data. The Kruskal–Wallis test was used for comparisons between intervention groups. The Mann–Whitney test was used for comparisons between two respective groups. The Wilcoxon signed-rank test was used to compare the measurements before and after rinsing with each intervention. The chi-square test was used to compare the percentage of plaque reduction between groups. The kappa test was used to evaluate the level of intraexaminer calibration. The level of statistical significance was considered at *p* < 0.05.

## 3. Results

The values of GCF influx and resting volume were not significantly different before and after rinsing with each intervention, respectively. However, the GCF influx values and resting volume decreased (0.81 µL/h to 0.72 µL/h and 0.0025 µL to 0.0020 µL, respectively) after rinsing with the combination, while they increased after rinsing with CHX and distilled water (Table 1).

Rinsing with combination induced a change in GCF influx (−0.086 ± 0.222; reduction), which was significantly different from changes induced after rinsing with CHX (0.088 ± 0.247; increase) and distilled water (0.075 ± 0.201; increase), as seen in Table 2. Meanwhile, the changes in GCF resting volume after rinsing with different interventions were not significantly different (Table 3).

**Figure 2:**
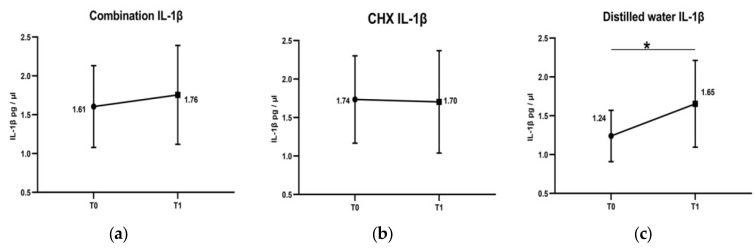
Changes of IL-1β concentrations in GCF after rinsing with combination, chlorohexidine (CHX) and distilled water. T0 is at baseline day 0. T1 is at the 4th day. After rinsing with combination, there was a non-significant slight elevation in the concentration of IL-1β (**a**). Rinsing with chlorohexidine (CHX) almost induced no changes in the concentration of IL-1β (**b**). Whereas, concentration of IL-1β was significantly increased after rinsing with distilled water (**c**). * Comparison by Wilcoxon signed-rank test, significance at *p* < 0.05.

Rinsing with both the combination (from 1.61 pg/µL to 1.76 pg/µL) and CHX (from 1.74 pg/µL to 1.7 pg/µL) induced no significant changes in the concentration of IL-1β despite plaque accumulation. However, a significant increase in the concentration of IL-1β, from 1.24 pg/µL to 1.65 pg/µL, was detected after rinsing with distilled water (see Figure 2).

**Figure 3:**
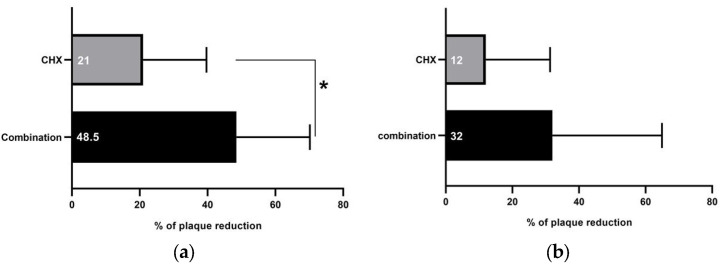
Percentage of plaque reduction after rinsing with combination and 0.12% chlorohexidine (CHX) mouthwashes. (**a**) At teeth sites where samples for GCF influx and resting volume assessments were taken, rinsing with combination mouthwash resulted in significantly more plaque reduction (48.5%) than CHX (21%). (**b**) At teeth sites where samples for IL-1β concentration assessments were taken, rinsing with both combination and CHX induced plaque reduction (32% and 12%, respectively). * Comparisons by Chi-square test; significance at *p* < 0.05.

The dental plaque accumulated on teeth surfaces was measured after rinsing with the study’s interventions in the three groups. At teeth sites where samples for GCF influx and resting volume assessments were taken, rinsing with the combination resulted in (mean PI = 2.53 ± 1.06) 48.5% less accumulated plaque than distilled water (mean PI = 4.93 ± 0.35), an anti-plaque effect that was significantly better than that observed with CHX (21% reduction; mean PI = 3.9 ± 0.87), as shown in Figure 3a. At teeth sites where samples for IL-1β concentration assessments were taken, less plaque was detected after rinsing with the combination (32%; mean PI = 3.4 ± 0.42), followed by CHX (12%; mean PI = 4.4 ± 0.96) compared to distilled water (mean PI = 5 ± 0.0), as shown in Figure 3b. Upon clinical examination of the oral cavity after rinsing with each intervention, no adverse effects were detected.

## 4. Discussion

The main finding of this study was the non-significant change in GCF influx accompanied with minimal fluctuations in GCF IL-1β levels after rinsing with the combination despite refraining from toothbrushing for 4 days. Interestingly, the combination induced a reduction in GCF influx. This finding was contrary to those observed in the CHX and distilled water groups (increase). In the literature, measurement of GCF was considered an early sensitive indicator of gingival inflammation [19]. An established relationship between an increase in GCF measurements and an increase in the degree of inflammation is well documented [22,23,24,25]. The tested combination is composed of two plant-derived extracts, green tea and *Salvadora persica* L. Green tea has been reported to have an anti-gingivitis effect that might be attributed to its catechin content [26,27]. Comparably, *Salvadora persica* L. extract has been found to clinically decrease periodontal inflammation and significantly reduce proinflammatory markers in GCF, such as IL-6 and TNF-α [15,28]. Therefore, rinsing with the combination might minimize the early inflammatory changes during this short-term period of no toothbrushing. This scenario is supported by the significantly better antiplaque effect elicited by the combination, a finding consistent with previous studies [13,14], taking into consideration that dental biofilm is the main cause of gingival inflammation [10].

However, the increase in the GCF influx after rinsing with CHX can be attributed to the fact that CHX could exhibit profound antiplaque properties only after meticulous removal of dental plaque guaranteed by toothbrushing [29]. Therefore, it might have lost its efficacy in reducing plaque accumulation during the non-brushing period of this study. Consequently, a relatively high quantity of plaque was allowed to develop, which in turn induced more inflammatory changes, resulting in a high GCF influx close to that of the distilled water group. Conversely, the resting volume values before and after rinsing with each particular intervention showed no significant difference. This result is in accord with expectations, as the resting volume was anticipated to remain unchanged because it is dependent on the depth of the crevicular sulcus, which is not expected to change considerably within 4 days [10,19].

As illustrated in distilled water group, GCF flow was not significantly changed, while GCF IL-1β levels were significantly elevated after 4 days of the non-toothbrushing period. As an interpretation, refraining from oral hygiene measures for 4 days was not enough to induce clinical signs of inflammation in gingival tissue. GCF flow is an early sensitive indicator of gingival inflammation [19], whereas, elevated GCF IL-1β levels means that inflammatory events have started in the gingival tissue in response to accumulated dental biofilm preceding the clinical signs of inflammation. Interestingly, both combination and CHX mouthwashes induced no significant elevation in GCF IL-1β levels after 4 days without toothbrushing. Therefore, both interventions might have the potential to retard the initiation of gingival inflammation.

Dental plaque accumulation on tooth surfaces is the main cause of gingival inflammation [10], which consequently increases GCF IL-1β concentrations. In this study, rinsing with the combination induced no significant changes in the concentration of GCF IL-1β, despite the presence of accumulated plaque. An increased concentration of IL-1β in the GCF is considered an early sensitive indicator for gingival inflammation [9], as noted after rinsing with distilled water. In the literature, frequent intake of flavonoid-rich food is accompanied by reduced salivary IL-1β and better periodontal health [30]; catechins are examples of flavonoids that are abundant in green tea [31]. Catechins, particularly (−)-epigallocatechin-3-gallate, were reported to reduce the neutrophil response and IL-1β and TNFα release in inflamed tissue [32]. Moreover, using aqueous extracts of green tea as irrigation was reported to reduce inflammation and suppress the level of IL-1β in gingival tissue [31]. Moreover, application of *Salvadora persica* L. extracts as a gel [33] or toothpaste [34] was reported to adjunct subsiding gingival inflammation [33]. Flavonoids identified in *Salvadora persica* L. extracts were reported to exert anti-inflammatory activity and to significantly reduce IL-1β release [35]. Thus, the combination might have anti-inflammatory effects attributed to the constituents of both green tea and *Salvadora persica* L. extracts. Such an effect may cause non-significant changes in GCF IL-1β concentration, comparable to the outcome observed after rinsing with the positive control CHX. This study revealed that the combination induced no significant changes in GCF flow and IL-1β levels for a short period of 4 days without oral hygiene measures. The study sample used subjects with healthy gingiva. This finding should not be generalized to patients suffering from gingivitis or periodontitis until further studies are conducted.

In the literature, the safety and biocompatibility of green tea and *Salvadora persica* L. aqueous extracts were extensively investigated. In animal studies, it has been reported that the intake of green tea up to 2500 mg/kg/day [36] as well as 400 mg/kg/day of *Salvadora persica* L. [37] are safe and exert no toxic effects after kidney and liver functions and hematological assessments. However, no previous study regarding the toxicity of their combination has yet been conducted. It is highly suggested to investigate the effects of this combination on cell viability, proliferation and apoptosis using cell lines culture as previously reported [38]. It is worth mentioning that this combination was topically used as a mouthwash in this study at concentrations far below the reported toxic thresholds of both extracts. Therefore, the authors assumed that oral rinsing with this mouthwash was safe.

The subjects that participated in this study were dental students who were blinded to the allocated interventions. One limitation of this study was that dental students might be aware about the bitter test of CHX, which consequently impaired the blindness. It was better to involve participants other than dental students with acquired dental knowledge in this study. However, the main outcomes in this study were GCF flow and IL-1β levels which were estimated objectively in site-specific protocol without any influence of the participants. Another limitation was that the authors used a sensitive scale to estimate the amount of fluid collected in the paper strips. Such measurements may have the disadvantage of increased variation in the recorded volumes as a result of fluid evaporation while measuring the volume. Nevertheless, this drawback was properly managed by the standardized very short distance between the subjects and the sensitive scale as well as the prompt weighing of the strips. Moreover, this study lacks a bacterial assessment of the accumulated biofilm, which could have provided a picture of the quality of the biofilm. Such assessment might be helpful in comparing the outcomes after rinsing with the interventions. The authors suggest this topic to be considered in future studies. Another possible limitation is the lack of measurements of bleeding on probing as a clinical parameter for gingival inflammation. However, the authors considered GCF measurements as a more sensitive parameter reflecting the degree of inflammation due to the relatively short-term duration of rinsing with interventions. Furthermore, GCF IL-1β was the only biomarker measured in this study. Possibly, it was better to include more biomarkers that participate in the innate immunity in the gingival tissue. However, the levels of GCF IL-1β are significantly elevated when patients refrain from toothbrushing for three days. This molecular event precedes the appearance of clinical signs of inflammation in the surrounding gingiva. Therefore, an elevated GCF IL-1β is considered an early sensitive indicator for gingival inflammation [9].

## 5. Conclusions

Despite the limitations of this study, rinsing with the combination mouthwash for a short-term (4 days) period in the absence of oral hygiene measures could potentially induce no significant change in GCF flow and less elevation in GCF IL-1β concentration despite the presence of plaque. Rinsing with the combination appears to have a beneficial effect on the health of gingival tissue and might have the potential to retard the initiation of gingival inflammation during undisturbed plaque accumulation on tooth surfaces for a relatively short period of time (4 days). Thus, it might potentially be considered in the production of natural oral healthcare products.

## Figures and Tables

**Table 1:** Estimation of GCF influx (fi), resting volume (Vr) and the time needed to form 1 mL (T) before and after rinsing with interventions.

		Mean V1 (µL)	Mean V2 (µL)	fi (µL/s)	fi (µL/min)	fi * (µL/h)	Vr ** (µL)	T (min)
Combination	before	0.009	0.007	0.00022	0.013	0.81	0.0025	4.5
	after	0.008	0.006	0.00020	0.012	0.72	0.0020	5.0
Chlorhexidine	before	0.008	0.006	0.00019	0.011	0.71	0.0028	5.2
	after	0.010	0.007	0.00022	0.013	0.80	0.0033	4.5
Distilled water	before	0.009	0.007	0.00020	0.012	0.72	0.0028	4.7
	after	0.009	0.007	0.00022	0.013	0.79	0.0032	4.5
Formula				V2/33	60 *fi (µL/s)	3600 *fi (µL/s)	V1-30 *fi	1/fi

* No significant statistical change in GCF influx before and after rinsing with each intervention; comparison by Wilcoxon signed-rank test, significance at *p* < 0.05; ** No significant statistical change in GCF resting volume before and after rinsing with each intervention; comparison by Wilcoxon signed-rank test, significance at *p* < 0.05.

**Table 2:** GCF influx changes (influx after–influx before) after rinsing with different study interventions.

	Mean Change ± SD	95% Confidence Interval		Versus	*p* Value *	Effect Size **	Power **
		Lower	Upper				
Combination	−0.086 ± 0.222	−0.193	0.021	CHX	<0.05	0.74	0.81
				distilled water	<0.05	0.76	0.81
Chlorhexidine	0.088 ± 0.247	−0.024	0.201	Combination	<0.05	0.74	0.81
				distilled water	>0.05	0.06	0.80
Distilled water	0.075 ± 0.201	−0.046	0.197	Combination	<0.05	0.76	0.81
				CHX	>0.05	0.06	0.80

* Comparisons by Mann–Whitney test; significance at *p* < 0.05. ** Calculated using G * Power software (version 3.1.9.2).

**Table 3:** GCF resting volume changes (resting volume after–resting volume before) after rinsing with different study interventions.

	Mean Change ± SD	95% Confidence Interval		*p* Value *
		Lower	Upper	
Combination	−0.0005 ± 0.0017	−0.0014	0.0003	>0.05
Chlorhexidine	0.0004 ± 0.0029	−0.0010	0.0017	
Distilled water	0.0004 ± 0.0025	−0.0011	0.0018	

* Comparison by Kruskal–Wallis test; significance at *p* < 0.05.

## Data Availability

Not applicable.
